# Use of DOOR and Win-Ratio Analysis in a Secondary Evaluation of a Histoplasmosis Clinical Trial

**DOI:** 10.1093/ofid/ofag089

**Published:** 2026-02-26

**Authors:** Tarsila Vieceli, Biyue Dai, Diego R Falci, Daiane Dalla-Lana, Cassia S M Godoy, Renata B A Soares, Monica B Bay, Hareton Vechi, Terezinha M J S Leitao, Lisandra S Damasceno, Marineide G Melo, Nathan C Bahr, David R Boulware, Alessandro C Pasqualotto

**Affiliations:** Post-graduation Program in Pathology, Federal University of Health Sciences of Porto Alegre, Porto Alegre, Brazil; Division of Biostatistics and Health Data Science, School of Public Health, University of Minnesota, Minneapolis, Minnesota, USA; Infectious Diseases Service, Hospital de Clínicas de Porto Alegre, Porto Alegre, Brazil; Post-graduation Program in Pathology, Federal University of Health Sciences of Porto Alegre, Porto Alegre, Brazil; Infectious Diseases Service, Hospital de Doenças Tropicais, Goiânia, Brazil; Infectious Diseases Service, Hospital de Doenças Tropicais, Goiânia, Brazil; Department of Infectious Diseases, Federal University of Rio Grande do Norte, Natal, Brazil; Department of Infectious Diseases, Federal University of Rio Grande do Norte, Natal, Brazil; Department of Community Health, Faculty of Medicine, Federal University of Ceará, Fortaleza, Brazil; Department of Community Health, Faculty of Medicine, Federal University of Ceará, Fortaleza, Brazil; Infectious Diseases Service, Hospital Nossa Senhora da Conceição, Porto Alegre, Brazil; Division of Infectious Diseases and International Medicine, Department of Medicine, University of Minnesota Medical School, Minneapolis, Minnesota, USA; Division of Infectious Diseases and International Medicine, Department of Medicine, University of Minnesota Medical School, Minneapolis, Minnesota, USA; Post-graduation Program in Pathology, Federal University of Health Sciences of Porto Alegre, Porto Alegre, Brazil; Infectious Diseases Service, Santa Casa de Porto Alegre, Porto Alegre, Brazil

**Keywords:** histoplasmosis, noninferiority trial, randomized clinical trial, statistical data analysis

## Abstract

Hierarchical composite end points may better reflect patient outcomes, rather than mortality alone. We applied DOOR and Win-Ratio analyses to a phase II trial comparing single high-dose vs standard liposomal amphotericin B for HIV-associated histoplasmosis. No clear differences were observed, supporting feasibility and informing design of future phase III trials.

## Teaser

Using DOOR and Win-Ratio methodologies to integrate mortality, toxicity, and rehospitalization into hierarchical composite endpoints, we compared single high-dose versus standard 14-day liposomal amphotericin B for HIV-associated disseminated histoplasmosis. The results demonstrated comparable overall clinical desirability between regimens, supporting the clinical viability of simplified induction strategies and paving the way for definitive phase III evaluation.

Histoplasmosis imposes a disproportionate burden on patients with advanced HIV disease (AHD). In low- and middle-income countries, case fatality rates in severe disease exceed 30% despite rollout of HIV therapy [1]. The World Health Organization (WHO) standard-of-care (SOC) induction regimen is liposomal amphotericin B (L-AmB) 3 mg/kg daily for 2 weeks. This remains prohibitive in many low-resource settings, where amphotericin B deoxycholate is used for induction therapy [2, 3].

To address these challenges, a phase II trial evaluated whether a single dose of liposomal amphotericin B (L-AmB) was noninferior to the standard L-AmB regimen [4]. Participants were randomized 1:1:1 to a 10-mg/kg single dose of L-Amb, a multiple-dose regimen (10 mg/kg on day 1 and 5 mg/kg on day 3), or SOC (3 mg/kg given for 2 weeks). Phase II results showed that single-dose L-AmB can be safely used as induction therapy in this patient population, supporting a phase III trial (clinicaltrials.gov NCT05814432) comparing a single 10-mg/kg dose with SOC. Traditional trials using single outcomes such as mortality or hospitalization do not capture trade-offs between efficacy and toxicity and often require large sample sizes when events are rare. When multiple events are of interest, combining end points into a composite assigns equal weights to outcomes that may not have the same importance. Hierarchical composite end points integrate multiple outcomes into one metric based on importance. Regulatory bodies endorse these approaches across cardiovascular, oncology, and antimicrobial trials [5], underscoring extension to neglected mycoses.

Two hierarchical composite end point–based approaches have been developed. The Win-Ratio statistic analyzes time-to-event outcomes in hierarchical order [6]. Each participant in the treatment group is compared with each participant from the control group to determine a win, loss, or tie, based on importance and timing. The treatment effect is estimated as the ratio of wins to losses; a ratio of >1 favors the treatment arm. The Win-Ratio is a relative metric and does not provide an absolute scale. A second methodology is desirability of outcome ranking (DOOR) [7], where participants are classified into several mutually exclusive categories ordered by clinical importance. Better DOOR probability >0.50 favors the treatment arm on a 0–1 probability scale. The distribution of the DOOR categories can be summarized for each arm, serving as an absolute scale.

Using phase II data, we applied DOOR and Win-Ratio analyses to compare SOC and single-dose L-AmB regimen across toxicity, rehospitalization, and mortality.

## METHODS

Consent, enrollment, and data collection have been previously described [4]. We developed a hierarchical end point using desirability of outcome ranking (DOOR) [6] to evaluate how a single-dose L-AmB (10 mg/kg) regimen compared with SOC induction (3 mg/kg daily for 2 weeks). Analyses used data from a completed phase II trial [4]. The 10 + 5-mg/kg arm was excluded because it performed similarly to the single-dose arm.

The DOOR hierarchy was developed a priori based on clinical severity, relevance, and consistency with DOOR applications. Outcomes were ranked hierarchically, with death as the worst outcome, followed by serious morbidity, treatment-related toxicity, and survival without major complications. The DOOR scale classified participants into 5 mutually exclusive categories (Table 1): DOOR category (1), death within 12 weeks; category (2), severe adverse events including rehospitalization within 2 weeks; category (3), grade 4 laboratory abnormality at 2 weeks; category (4), grade 3 laboratory abnormalities at 2 weeks; and category (5), alive at 12 weeks with no major toxicities. Participants lost to follow-up within 2 weeks were assigned DOOR category (1) (likely to have died from disseminated histoplasmosis), and after 2 weeks category (4). For laboratory abnormality grading, we used the Division of AIDS (DAIDS) severity scale [8], classifying patients by highest toxicity. The Wilcoxon rank-sum test was used for hypothesis testing comparing 2 arms, with better DOOR probability calculated. We conducted the sensitivity analysis using the Win-Ratio statistic [7]. For each pair, outcomes were evaluated sequentially according to the prespecified hierarchy in the DOOR framework, resulting in a win, loss, or tie. Component analyses were followed for both DOOR and the Win-Ratio to assess consistency of treatment effects. Matching was not performed because randomization ensured baseline comparability, and matching could reduce efficiency and introduce bias in small samples.

**Table 1.** Distribution of Patients According to DOOR Maximal Score in Single High-Dose Liposomal Amphotericin B vs Standard-of-Care Daily Liposomal Amphotericin for Two Weeks in HIV-Related Disseminated Histoplasmosis

Desirability of Outcome Ranking Categories, Maximal Rank^a^		Single High-Dose Amphotericin (n = 40), No. (%)	Standard-of-Care Daily Amphotericin B (n = 39), No. (%)
Category	Meaning	…	…
1	Death at 12 wk or loss of follow-up at 2 wk	10 (25)	8 (21)
2	Severe Adverse Event including rehospitalization at 2 wk	7 (18)	3 (8)
3	Grade IV laboratory abnormalities at 2 wk	3 (7)	2 (5)
4	Grade III laboratory abnormalities within 2 wk or loss of follow-up between wk 2 and wk 12	9 (22)	13 (33)
5	Alive at 12 wk without major adverse events	11 (28)	13 (33)

Desirability of Outcome Ranking Categories, Individual Events	Single High-Dose Amphotericin (n = 40), No. (%)	Standard-of-Care Daily Amphotericin B (n = 39), No. (%)
Death within 12 wk	10 (25)	7 (18)
LFU within 2 wk	0 (0.0)	1 (3)
LFU between 2 and 12 wk	1 (2)	1 (3)
SAE within 2 wk	3 (7)	2 (5)
Rehospitalization within 2 wk	4 (10)	1 (2.6)
No. of grade 4 laboratory abnormalities at 2 wk^b^	…	…
0	28 (70)	32 (82)
1	12 (30)	7 (18)
No. of grade 3 laboratory abnormalities within 2 wk^a^	…	…
0	28 (70)	26 (66)
1	11 (28)	12 (31)
2	0 (0)	1 (3)
3	1 (2)	0 (0)
Alive and without events above	11 (28)	13 (33)

Abbreviations: DOOR, Desirability of Outcome Ranking; LFU, loss to follow-up; SAE, severe adverse event.

^a^ *P* = .29 by Wilcoxon rank-sum test.

^b^Proportion of those who experienced at least 1 event in the category. Numbers in the first half of the table refer to maximum DOOR rank; the bottom half refers to the number of individual events.

This analysis is a secondary, post hoc evaluation of a completed phase II trial. DOOR and Win-Ratio analyses were prespecified but not used to inform trial design or sample size; therefore, no power calculation was performed for these analyses.

## RESULTS

A total of 118 participants were included in the phase II trial: 40 received a single 10-mg/kg L-AmB dose, 39 received 10 mg/kg +5 mg/kg, and 39 received SOC. As the single-dose arm performed similarly to 10 mg/kg D1 + 5 mg/kg D3 in the primary analysis [4], DOOR and Win-Ratio analyses focused on the single-dose and SOC arms.

**Figure 1.**
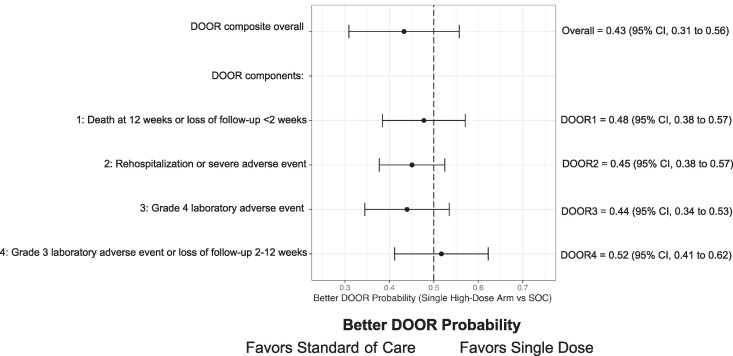
Forest plot for composite better door probability and individual components of the DOOR scale. The component-wise Better Door Probability was calculated marginally for each component. That is, a participant could appear in several categories. For each event type, the comparison models having the event (worse) vs not having the event (better). Data points located to the left of the vertical line at 0.50 probability indicate outcomes that favor the standard of care. Abbreviation: DOOR, desirability of outcome ranking.

We observed no statistical difference in clinical outcomes using DOOR or Win-Ratio. The probability of a favorable DOOR outcome for single-dose L-AmB vs SOC was 0.43 (95% CI, 0.31–0.56; *P* = .29) (Figure 1), favoring SOC. The Win-Ratio for single high-dose L-AmB vs SOC was 0.69 (95% CI, 0.37–1.27; *P* = .23) (Supplementary Figure 1).

In the DOOR analysis, participants were classified by maximal rank as DOOR category 1: 10 (25%) vs 8 (21%) in the single high-dose arm vs SOC, respectively; category 2: 7 (18%) vs 3 (8%); category 3: 3 (7%) vs 2 (5%); category 4: 9 (22%) vs 13 (33%); category 5: 11 (28%) vs 13 (33%).

## DISCUSSION

These analyses reinforce the phase II trial observation that a single high dose of L-AmB yields clinical outcomes comparable to the SOC for HIV-associated disseminated histoplasmosis. By integrating mortality, rehospitalization, toxicity, and laboratory abnormalities into a patient-centered composite end point, we build on previous findings showing that clinical benefit of the short-course strategy does not appear to be inferior in a small exploratory data set. Although the phase II trial demonstrated noninferiority of the single-dose regimen using a binary outcome, the DOOR-based analysis yields a point estimate numerically favoring standard of care, without difference between arms. This does not indicate contradiction between analyses but reflects the distinct objectives and properties of hierarchical end points in a small exploratory data set. Wide confidence intervals hinder definitive conclusions, and results should not be interpreted as evidence of inferiority or equivalence.

The DOOR framework is uniquely suited to multidimensional tradeoffs, weighting clinical end points against toxicity. DOOR is increasingly used in clinical trials and was developed for hospital-acquired and ventilator-associated bacterial pneumonia [9] and *Staphylococcus aureus* bacteremia [10]. The Win-Ratio is popular in cardiology trials evaluating multiple outcomes, as a hierarchy of components might reflect clinical priorities [11]. Both were utilized in a phase II trial of cryptococcal meningoencephalitis [12], and these methods have future relevance for noninferiority trials—where survival is but one aspect of overall patient experience.

Our data should be interpreted in the context of growing evidence that shortened L-AmB schedules can be safe and effective across conditions [13]. Beyond the phase II histoplasmosis trial [4], shorter courses were noninferior for cryptococcal meningoencephalitis [14] and visceral leishmaniasis [15] in large phase III trials, supporting biological plausibility of single-dose regimens, which offer compelling advantages, reducing costs and eliminating daily infusions and need for prolonged inpatient care. These benefits directly address access barriers that sustain the unacceptably high mortality of disseminated histoplasmosis.

Our analysis highlights practical challenges when applying DOOR or Win-Ratio methodology to small studies: Statistical power is limited, and wide confidence intervals underscore the risk of type II error. A higher rate of rehospitalization was observed in the single-dose arm. Given the small number of events and wide confidence intervals, this imbalance may reflect random variation rather than a true difference between regimens. The results were fragile, where 1–2 differences per arm could swing the DOOR probability; thus we caution against overinterpretation. Optimal categorization of missing data is unclear, particularly loss to follow-up after week 2; in our sample, no patients were lost to follow-up between weeks 2 and 12, and patients lacked other adverse events. Herein, we assigned DOOR category [4], but differential attrition between arms could bias results. Laboratory toxicity grading relied on 2-week assessments and did not capture the transient toxicity that may have occurred earlier.

Another limitation is that outcome assessment duration for DOOR components was constrained by the design and schedule of the parent phase II trial, particularly for rehospitalization; harmonization of time points is desirable, as rehospitalization could relate to inadequate induction later in follow-up. Measuring different time horizons may incompletely capture patient experience over follow-up. This reflects the structure of the original trial rather than analytic choices and may have influenced the relative weighting of toxicity vs later clinical events. Laboratory toxicities were assessed at week 2, at the end of induction therapy, whereas mortality and rehospitalization were evaluated over the 12-week follow-up period. This time point was chosen because laboratory events developing later are more likely attributable to other factors. Future trials with DOOR as a primary end point should prospectively harmonize outcome assessment windows to reflect longitudinal risk–benefit trade-offs.

Notwithstanding these limitations, this work adds to the rationale for an appropriately powered phase III trial (clinicaltrials.gov identifier: NCT05814432). DOOR and Win-Ratio are appropriate methodologies to evaluate noninferiority for both survival and toxicity.

## Supplementary Material

ofag089_Supplementary_Data

## Supplementary Data

Supplementary materials are available at *Open Forum Infectious Diseases* online. Consisting of data provided by the authors to benefit the reader, the posted materials are not copyedited and are the sole responsibility of the authors, so questions or comments should be addressed to the corresponding author.
