# Supplementary material for: Use of DOOR and Win-Ratio Analysis in a Secondary Evaluation of a Histoplasmosis Clinical Trial
Source: Open Forum Infect Dis. 2026 Feb 26;13(3):ofag089. doi: 10.1093/ofid/ofag089 (PMC12994473; doi:10.1093/ofid/ofag089)
Supplement: ofag089_Supplementary_Data [file ofag089_supplementary_data.zip › supplement 1501.doc]

**Supplemental Figure 1. Forest plot for Win-Ratio statistic comparing single dose amphotericin vs standard-of-care daily amphotericin B in HIV-related disseminated histoplasmosis.**

Alt text: graphical representation of the Win-Ratio for each component, showing no statistical differences between arms.
